# DNA Sequencing by Hexagonal Boron Nitride Nanopore: A Computational Study

**DOI:** 10.3390/nano6060111

**Published:** 2016-06-06

**Authors:** Liuyang Zhang, Xianqiao Wang

**Affiliations:** College of Engineering, University of Georgia, Athens, GA 30602, USA; lyzhang@uga.edu

**Keywords:** molecular dynamics simulation, hexagonal boron nitride, ssDNA sequencing

## Abstract

The single molecule detection associated with DNA sequencing has motivated intensive efforts to identify single DNA bases. However, little research has been reported utilizing single-layer hexagonal boron nitride (hBN) for DNA sequencing. Here we employ molecular dynamics simulations to explore pathways for single-strand DNA (ssDNA) sequencing by nanopore on the hBN sheet. We first investigate the adhesive strength between nucleobases and the hBN sheet, which provides the foundation for the hBN-base interaction and nanopore sequencing mechanism. Simulation results show that the purine base has a more remarkable energy profile and affinity than the pyrimidine base on the hBN sheet. The threading of ssDNA through the hBN nanopore can be clearly identified due to their different energy profiles and conformations with circular nanopores on the hBN sheet. The sequencing process is orientation dependent when the shape of the hBN nanopore deviates from the circle. Our results open up a promising avenue to explore the capability of DNA sequencing by hBN nanopore.

## 1. Introduction

DNA sequencing has been the focus of many researchers for several decades [1,2,3,4]. The recent development of entirely new strategies for DNA sequencing has reinvigorated this field. The fundamental idea is to simultaneously thread single-stranded DNA through the nanopore and detect the sequence-dependent ionic currents through the pore. DNA translocation through nanopores could cause a sudden drop in the ionic current due to the DNA occupation of the nanopores [5,6,7]. Such a DNA sequencing approach with nanopores provides a promising technology for fast DNA sequencing free of fluorescent labeling steps [8]. One major challenge faced by the nanopore-based sequencing is to achieve a high spatial resolution in order to distinguish the types of DNA bases, which requires the thickness of the nanopore to be comparable with the stacking distance of the DNA base pair.

Graphene, as a two-dimensional material, has been extensively studied both experimentally and theoretically due to its extraordinary mechanical, optical and electronic properties in recent years [9,10,11,12,13,14]. It has been demonstrated that nanopores fabricated from graphene sheets can be made extremely thin and structurally robust to allow the identification of single nucleotides, which opens a new chapter in DNA sequencing [15,16,17,18,19,20,21]. However the graphene-based nanopore suffers from significant signal noises. The reason is that graphene is highly hydrophobic and very sticky to the DNA strand, therefore trapping DNA bases during its translocation. This trap contributes to a high noise level for sequencing [15].

Hexagonal boron nitride (hBN), another type of two-dimensional material, has become a hot topic of research thanks to its structural equivalency to graphene and its outstanding mechanical [22], thermodynamic [23], and electronic properties [24]. Hexagonal boron nitride (hBN) is an atomic lattice structure similar to graphene, but composed of the heterogeneous atoms boron and nitrogen in place of carbon. hBN is less hydrophobic than graphene which can minimize the hydrophobic interaction that impedes the DNA translocation through the constructed nanopores. Moreover, the thickness of hBN is comparable to the spacing between nucleotides in (single-strand DNA) ssDNA (0.32–0.52 nm) [25]. It also shows other advantages over graphene in terms of its insulating property in high-ionic-strength solution and fewer defects made during the manufacturing process [26]. The fundamental properties of DNA nucleobases and hBN sheets remain unchanged upon adsorption, which suggests its promising application for DNA research [27]. The ultrathin hBN nanopores provide substantial opportunities in realizing high-spatial-sensitivity nanopore devices for various applications [26]. All above-mentioned properties suggest hBN to be a promising candidate for nanopore devices. However, the investigation of DNA sequencing by two-dimensional hBN is still lacking and is worthy of exploration.

To further evaluate the mechanism of hBN nanopores for DNA sequencing, here we report the study of ssDNA translocation through hBN nanopores using large-scale molecular dynamics (MD) simulations which can provide atomic details of the transport process [28]. We also investigate the binding interaction between the DNA base and hBN sheet as well as the influence of the geometry of the nanopore on the sequencing outcome. Elucidating the interaction between hBN and the DNA base is crucial for the design of next-generation hBN pore–based DNA sequencing devices.

## 2. Results and Discussions

**Figure 1:**
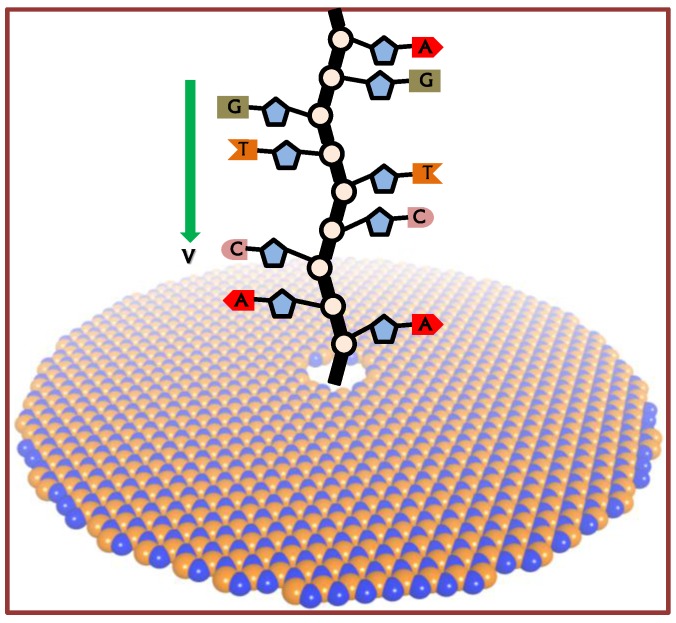
Scheme of single-strand DNA (ssDNA) sequencing with hexagonal boron nitride (hBN) nanopore. The ssDNA is placed right above the hBN nanopore and perpendicular to the hBN sheet. The red, orange, pink and grey areas represent the adenine (A), thymine (T), cytosine (C) and guanine (G) nucleotides separately.

The simulation system consists of the hBN sheet with a nanopore of specific geometry and a ssDNA molecule. Initially, the hBN sheet is placed in the *x*-*y* plane with the center of mass at the Cartesian coordinate origin (0,0,0). A circular nanopore is constructed by deleting the atoms with their coordinates satisfying *x*^2^ + *y*^2^ < *R*^2^, where *R* is the radius of the BN nanopore (*R* = 0.5 nm). In order to study the geometric effect of nanopores, an elliptical nanopore with the same area as the circular nanopore is also constructed. A model ssDNA molecule with the sequence AACCTTGGAACCTTGGAACCTTGG is introduced and energetically minimized before sequencing. The intentional ssDNA sequence with a repeated unit is to make sure the interaction energy profile is consistent for the same type of nucleotide. We can also use the other sequence with a random permutation or order; however, the sequence order will not affect the results. As shown in Figure 1, the ssDNA molecule is initially placed close to the nanopore.

### 2.1. Interaction between Nucleobases and hBN Sheet

**Figure 2:**
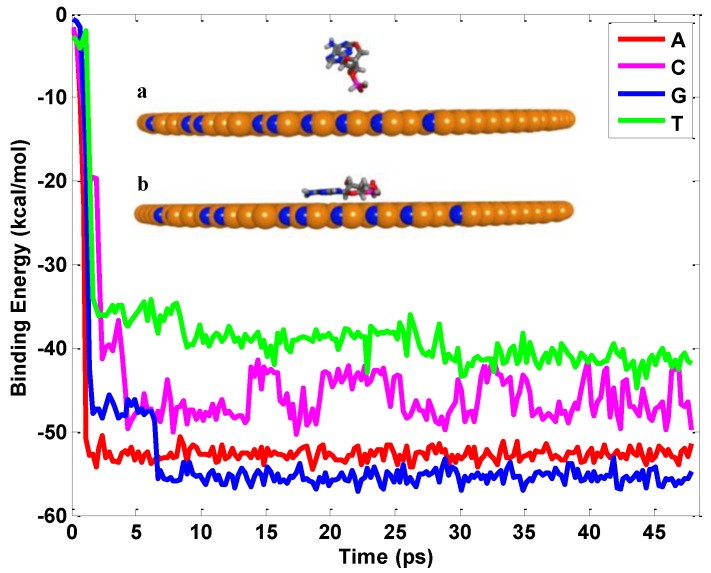
Evolution of binding energy between hBN and four basic nucleotides.

From density functional theory (DFT) calculations, it has been revealed that the hBN possessed high sensitivity for the nucleobases and has good potential in DNA detection biosensors [29,30]. The interfacial interaction between the single nucleobase and hBN sheet lays a critical foundation for the ssDNA sequencing. Here the binding energy on the interface of the hBN and ssDNA is characterized to distinguish different types of nucleobases. Each type of nucleobase is initially placed above the circular hBN sheet (diameter 8 nm) within the cutoff distance. Subsequently it adsorbs onto the hBN sheet due to the strong attraction force between them. For each type of nucleobase, different initial conditions are studied to ensure that the calculated interfacial energy does not depend on the initial conformations. If the simulation time were long enough, all the states of the interface could be explored. The magnitude of the intermolecular interaction energy provides a direct measure of the strength of the interfacial energy between the nucleobase and the hBN sheet, as shown in Figure 2. It can be seen that nucleotide G exhibits the strongest binding interaction of −55.5 kcal/mol with the hBN sheet compared to nucleotide A (−52.7 kcal/mol), nucleotide C (−45.8 kcal/mol) and nucleotide T (−41.2 kcal/mol), which can be attributed to different side groups of nucleobases. The initial and final configurations of nucleotide A are documented in Figure 2. Nucleotide A orientates its aromatic ring plane to align to the surface of the hBN sheet, and then moves closer to achieve a low potential energy status. All nucleobases exhibit the same stacking arrangement on the hBN sheet due to the polarization effect: the anions (N and O atoms) of nucleobases prefer to stay on top of the cations (B) of the BN sheet as far as possible, regardless of the biological properties of nucleobases [27].

**Figure 3:**
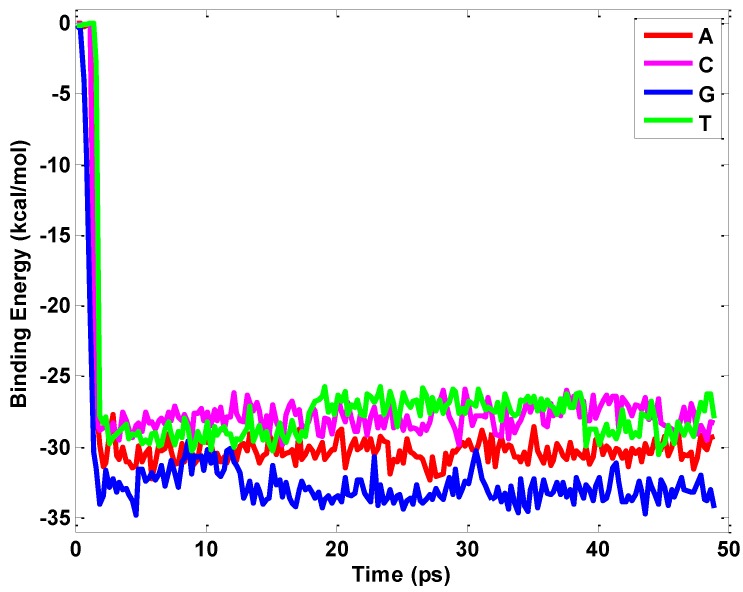
Evolution of binding energy between graphene and four basic nucleotides.

For comparison, Figure 3 shows the interfacial energy profile between the nucleobase and a graphene sheet. Nucleotide G exhibits the strongest interaction of −33.28 kcal/mol with the graphene sheet compared to nucleotide A (−30.32 kcal/mol), nucleotide C (−28.16 kcal/mol) and nucleotide T (−27.9 kcal/mol). The magnitudes of the interaction energies of the nucleobases with graphene are similar to those found with single-walled carbon nanotubes [31,32,33,34]. By isothermal titration calorimetry [35], the relative interaction energies of the nucleobases with graphene have been found to decrease in the order G > A > C~T in aqueous solution. Our simulations observe the same trend that the graphene sheet shows while the strength between them is significantly high [31,32,36,37]. Compared with the hBN sheet, the binding interaction between the graphene and nucleobase are only from the van der Waals (vdW) interaction. Except for the van der Waals interaction between the hBN sheet and nucleobase, the electrostatic interaction is also involved for the interfacial interaction. Graphene is a gapless semimetal with the nonpolar nature of the homonuclear C–C bond, while the BN sheet is an insulator with a polar nature underlying the charge transfer between its constituent B and N atoms [30]. The difference in electronegativity between B and N results in a combination of weak ionic and covalent bonding that is quite distinct from neutral graphene sheets [38,39].

**Figure 4:**
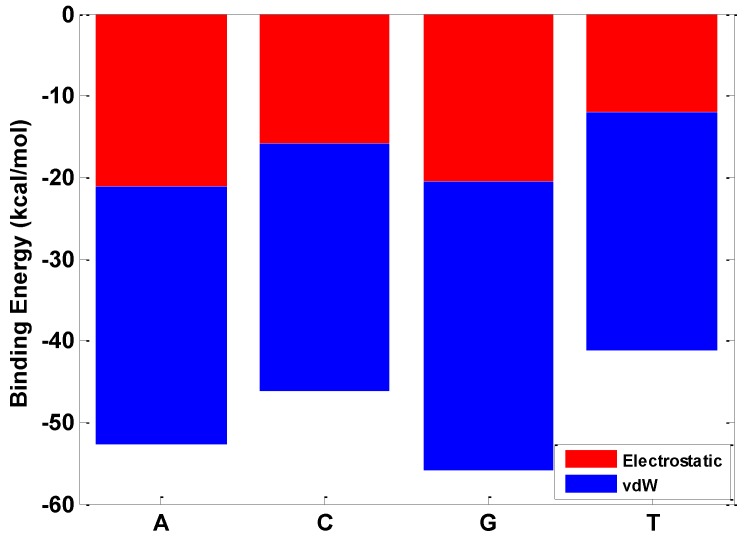
Contribution of van der Waals (vdW) and electrostatic interaction in the binding energy between hBN and four basic nucleotides.

The electrostatic interaction plays an important role in the interfacial interaction between the BN sheet and nucleobase, as shown in Figure 4. The electrostatic interaction contributes to the interfacial interaction between the nucleobases of A, C, G, T and hBN which are −21.2 kcal/mol, −15.8 kcal/mol, −20.5 kcal/mol, and −12.1 kcal/mol, respectively. Besides, based on the interfacial energy profile of nucleotide C and T, the hBN sheet presents higher sensitivity to the nucleobase than graphene and could better differentiate the nucleotides C and T. The difference of the interfacial interaction between the hBN sheet and the nucleobase could distinguish nucleotides A, T, C, G from one another and acts as a scalable powerful measurement parameter for ssDNA sequencing.

### 2.2. ssDNA Aligning

**Figure 5:**
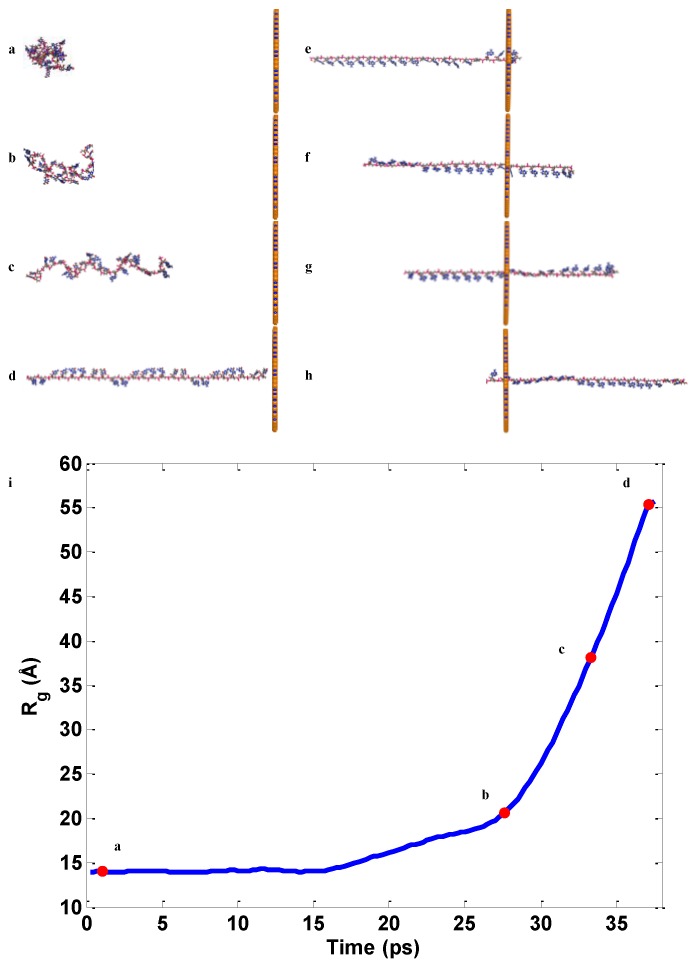
Conformation of stretching process of ssDNA before passing through the hBN nanopore (**a**–**d**); Evolution of real-time process of passing-through the hBN nanopore (**e**–**h**); Evolution of real-time radius of gyration (**i**).

The aligning and sequencing process of ssDNA translocation through the circular BN nanopore is shown in Figure 5. After energy minimization, the ssDNA forms a cluster structure due to the hydrogen bonding, e.g., A-T, C-G, which makes it difficult to uncouple the nucleobases from one another. Also, the ssDNA cluster is easy to stick to the surface of the hBN sheet due to the appreciable van der Waals and electrostatic interfacial interactions. The sticking phenomenon causes the low accuracy of detecting different nucleobases. Thus, the aligning process of ssDNA is critical to the sequencing and increases the detection accuracy of the nucleobase. It is unavoidable and extremely necessary to separate each nucleobase before passing through the nanopore. In our simulations, one nucleotide on the one end of ssDNA is selected and pulled out from the ssDNA cluster as shown in Figure 5a–d. To clarify the aligning process of nucleobases before passing the BN nanopore, the radius of gyration *R*_g_ of all atoms of ssDNA is calculated as [40,41],

(1) $$R_{g}=\sqrt[{2}]{\frac{1}{n}\sum |r_{i}^{2}-r_{\text{com}}^{2}|}$$

where *n* is the number of atoms, *r_i_* is the position of the center of mass of the *ith* atom and *r*_com_ is the position of the center of mass of the ssDNA. At the beginning, with the minimum value of *R*_g_ = 14.04 Å, the ssDNA keeps a stable cluster state. The increase of *R*_g_ indicates that the ssDNA cluster is uncoupled and the ssDNA is aligned into a thread-like structure. The *R*_g_ with a large value of 55.68 Å indicates that the ssDNA reaches a sparse distribution, seen here as a thread-like distribution. The nucleobase is uniformly distributed along the thread-like structure.

### 2.3. ssDNA Sequencing

With the thread-like ssDNA, the center of mass of the first nucleotide is chosen to pull through the hBN nanopore at a constant velocity of 0.1 Å/ps. The hBN nanopore with a diameter ~1 nm corresponds to the optimal spatial resolution by geometry-mapping curves from another researcher’s work [26]. It also agrees with the geometric diameter of nanopores obtained from TEM characterization of the pore by considering the stern layer or immobilized hydrates’ layer thickness on the pore and solution interface [42]. The basic nucleotide is found to translocate through the nanopore in a similar way with the translocation process exhibiting a base-by-base ratcheting fashion [43] that could significantly slow down the ssDNA translocation and extends the DNA detection time. In order to increase the detection accuracy, the simulation is repeated five times for each simulation system.

**Figure 6:**
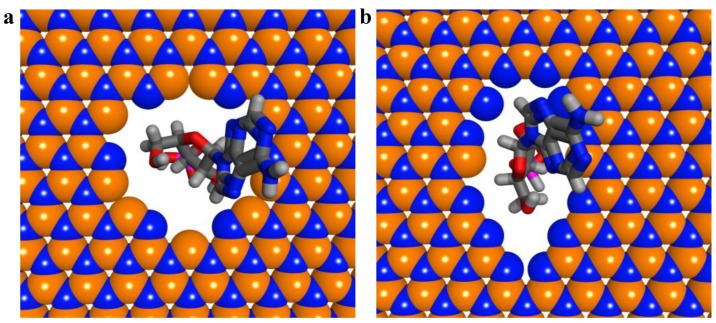
Hexagonal boron nitride (hBN) nanopores of different geometries and illustration of nucleotides passing through the nanopore: (**a**) circular nanopore; (**b**) elliptical nanopore.

It has been demonstrated that the shape of nanopores in graphene plays a vital role in the accuracy of DNA sequencing [44]. To investigate the effect of nanopore geometry in the hBN sheet on sequencing, circular and elliptical nanopores with same area are constructed on the BN sheet, respectively, as shown in Figure 6.

**Figure 7:**
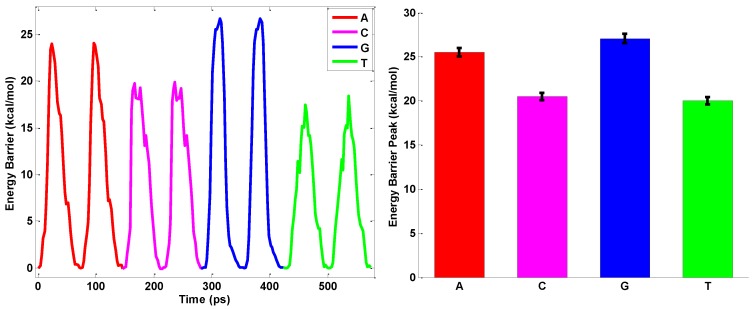
Energy profile when ssDNA passed through the circular hBN nanopores. The energy peak values for different nucleotides are extracted from the energy profile.

**Figure 8:**
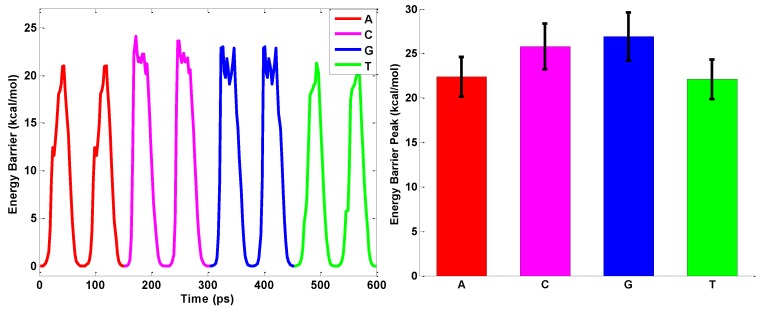
Energy profile when ssDNA passes through the elliptical hBN nanopores. The energy peak values for different nucleotides are extracted from the energy profile.

Figure 7 and Figure 8 depict the profile of interaction energy when the ssDNA threads the circular and elliptical nanopores, respectively. As shown in Figure 7, the magnitudes in the ssDNA translocation through the nanopores imply that each nucleobase threading the BN nanopore can be read off from the profile of interaction energy directly. Each nucleotide threading the nanopore undergoes a significant conformational change that explains the change of the magnitude of interaction energy when the nucleobase passes through the nanopore. With the circular hBN nanopore, the characteristic magnitude peaks for the two pyrimidine bases (C and T) are lower than those of the purine bases (A and G). The different components of nucleobases make the volume of the purine bases larger than that of pyrimidine bases and lead to the difference in the characteristic peaks of binding energy when the ssDNA passes through the nanopore [44]. The binding energy peak values averaged over the same nucleotides from 10 simulations are calculated and are shown in Figure 7. The magnitude peaks of interaction energy of nucleotides A, T, C, and G are 25.5 kcal/mol, 20 kcal/mol, 20.5 kcal/mol, and 27.06 kcal/mol, respectively. The magnitude of energy peak decreases with the order G > A > C > T following the trends of interfacial interaction between the BN sheet and nucleobases. From the binding energy peaks, we can find that the nucleotides A, T, C, G in the ssDNA could be distinguished from one another with the circular hBN nanopore. The interaction energy profile observed in our simulation agrees with the force profile obtained from the ssDNA sequencing by the graphene nanopore [44]. From the experimental viewpoint, the interaction energy between the nucleobase and two-dimensional layers has a correlation with the conductance which shows a means to differentiate the four nucleobase types [45]. For example, the conductance fluctuation of pyrimidines is found to be much larger than that of purines, resulting from the interaction strength between the nucleobase and the nanopore.

As seen from Figure 8, the interaction energy profile for different nucleobases with the elliptical nanopore is different from that of the circular nanopore. As discussed in ssDNA sequencing by rhombic pore on graphene [44], the conformation of the nucleotide with the phosphodiester bond is narrow which makes it easier for the nucleotide to pass through the nanopore along the major axis than along the minor axis of the elliptical nanopore. It has been recorded that the conformational change of the nucleotide is quite different when threading the nanopore in various orientations [17,46]. The effective area of the elliptical nanopore for the ssDNA to pass through is smaller as is the direction along the minor axis of the elliptical nanopore, which makes it extremely difficult for the base to thread the nanopore. The ratcheting-fashion could increase the energy peaks when the orientation of the nucleobase changes; therefore, the elliptical nanopore could not guarantee the correct discrimination of different types of nucleotides. However, the circular nanopore is axisymmetric and the energy peak of a nucleobase through the nanopore is almost orientation-independent. The axisymmetric circular nanopore is more robust to the orientation of the nucleobase than the asymmetrically elliptical nanopore. The observed difference of the binding energy further verifies the fact that deoxyribonucleic acid (DNA) sequencing is very sensitive to the orientation of the nucleotide when the geometry of the nanopore is asymmetrical [47]. The basic nucleotides (A, T, C, G) can be identified by the interaction energy peaks only with the circular hBN nanopore and the magnitude of the energy peaks is dependent on the type of nucleobase, which means that bases of different geometries could be identified with the hBN nanopore.

## 3. Methods

In molecular dynamic (MD) simulations, we adopt the modified CHARMM27 force field [48] to describe the bonded and non-bonded interactions between atoms. The potential components described in the CHARMM27 force field are defined as the intramolecular interactions (bond stretch, bond angle, dihedral angle, improper angle, Urey-Bradley) characterizing the short-range bonding and intermolecular non-bonded interactions describing the long-range van der Waals (vdW) interactions and electrostatic interactions. The non-bonded part is computed as a sum of Coulomb and Lennard-Jones contributions for pairwise intra- and inter-molecular interactions related to electrostatic and van der Waals interactions:

(2) $$E_{\text{nonbonded}}=\underset{i<j}{\sum }\left[\frac{q_{i}q_{j}}{r_{ij}}+4\varepsilon_{ij}\left(\frac{\sigma_{ij}^{12}}{r_{ij}^{12}}-\frac{\sigma_{ij}^{6}}{r_{ij}^{6}}\right)\right]$$

where *r_ij_* is the distance between two atoms *i* and *j*, ε*_ij_* is the depth of the potential well, and σ*_ij_* is the distance at which the potential becomes zero. *q_i_* and *q_j_* are the charge on atom *i* and *j*. The summation runs over all of the pairs of atom *i* < *j* on molecules A and B or A and A for the intramolecular interactions. For the Lennard-Jones (LJ) potential, Lorentz-Berthelot mixing rules for the Lennard-Jones coefficients are employed: σ*_ij_* = (σ*_ii_* + σ*_jj_*)/2 and $\varepsilon_{ij}={\left(\varepsilon_{ii}\varepsilon_{jj}\right)}^{1/2}$. For hBN, σ_B_ = 3.453 Å, σ_N_ = 3.365 Å and ε_B_ = 4.16 meV, ε_N_ = 6.281 meV [49,50]. The partial charges on hBN are taken from previous density functional theory calculations [38,51]. In our MD simulation, *q*_B_ = 0.37e, *q*_N_ = −0.37e, $q_{H-B}=0.32e$, $q_{H-N}=-0.19e$. Boron atoms with a lower electronegativity compared to the nitrogen atoms are positively charged while the nitrogen atoms are negatively charged in B–N bonding. H atoms forming dangling bonds with B and N atoms at the edge of the sheet have either positive or negative charges depending on the atoms to which the H atoms bind. Energy minimization based on the conjugated gradient algorithm is performed to find the thermally stable configuration and achieve a conformation with a minimum potential energy for the system. After the equilibrium state is achieved, canonical ensemble (constant-number, constant-volume, constant-temperature) simulations with temperature 300 K are carried out based on the Berendsen thermostat [52]. The velocity Verlet time stepping method is utilized with the integration time step 0.5 fs. A cutoff distance of 10 Å is used for all potentials. All simulations are performed in the generalized born implicit solvent environment [53]. To speed up computation, the hBN atoms are fixed to their initial position which facilitates the geometrical characterization of the ssDNA with respect to the hBN. The global cutoff for the LJ term is set here to be 10 Å as a good balance between computational cost and accuracy.

## 4. Conclusions

In summary, here we have performed molecular dynamics simulations to investigate the mechanism of ssDNA sequencing using the hBN nanopore. We first demonstrate the interfacial interaction between the hBN sheet and a single nucleobase which lays the foundation for hBN-based ssDNA sequencing. With strong vdW and electrostatic interactions, the nucleobase can fully interact with the hBN sheet and attach onto its surface. The simulation results demonstrate that nucleotide G has the strongest binding strength compared with other nucleobases. The type of nucleobase could be classified and identified from the binding strength between the nucleobase and hBN sheet. Compared with the binding strength between graphene and the nucleobases, our result implies that the effect of hBN-based ssDNA sequencing is more sensitive than graphene-based ssDNA sequencing. The ability of nucleobase detection by the hBN nanopore depends both on the binding strength between the nucleobase and hBN sheet and the nanopore geometry. Our simulation suggests that a circular nanopore on the hBN sheet is better suited to DNA sequencing than an asymmetric nanopore. These fundamental findings from the interfacial binding strength between hBN and nucleobases provide promising guidance for designing novel hBN-based devices for biological applications.
